# DNA Profiling of Spermatozoa by Laser Capture Microdissection and Low Volume-PCR

**DOI:** 10.1371/journal.pone.0022316

**Published:** 2011-08-11

**Authors:** Cai-xia Li, Jun-ping Han, Wen-yan Ren, An-quan Ji, Xiu-lan Xu, Lan Hu

**Affiliations:** 1 Institute of Forensic Science, Ministry of Public Security, Beijing, China; 2 Key Laboratory of Forensic Genetics, Ministry of Public Security, Beijing, China; 3 Chinese Peoples Public Security University, Beijing, China; Tor Vergata University of Rome, Italy

## Abstract

Genetic profiling of sperm from complex biological mixtures such as sexual assault casework samples requires isolation of a pure sperm population and the ability to analyze low abundant samples. Current standard procedure for sperm isolation includes preferential lysis of epithelial contaminants followed by collection of intact sperm by centrifugation. While effective for samples where sperm are abundant, this method is less effective when samples contain few spermatozoa. Laser capture microdissection (LCM) is a proven method for the isolation of cells biological mixtures, even when found in low abundance. Here, we demonstrate the efficacy of LCM coupled with on-chip low volume PCR (LV-PCR) for the isolation and genotyping of low abundance sperm samples. Our results indicate that this method can obtain complete profiles (13–16 loci) from as few as 15 sperm cells with 80% reproducibility, whereas at least 40 sperm cells are required to profile 13–16 loci by standard ‘in-tube’ PCR. Further, LCM and LV-PCR of a sexual assault casework sample generated a DNA genotype that was consistent with that of the suspect. This method was unable, however, to analyze a casework sample from a gang rape case in which two or more sperm contributors were in a mixed population. The results indicate that LCM and LV-PCR is sensitive and effective for genotyping sperm from sperm/epithelial cell mixtures when epithelial lysis may be insufficient due to low abundance of sperm; LCM and LV-PCR, however, failed in a casework sample when spermatozoa from multiple donors was present, indicating that further study is necessitated.

## Introduction

Genetic profiling in forensic science often requires the isolation of a population of cells from complex biological mixtures. Sexual assault cases are the most frequent type of case submitted to crime labs, and often contain mixed samples of male and female DNA. When ample sperm is present in a sample, spermatozoa are isolated by preferential lysis of epithelial cells present in the sample. Sperm can then be separated from the cell debris by centrifugation. In azoospermic sexual assault cases, The Forensic Science Service applies Fluorescence in situ hybridization (FISH) and laser microdissection (LMD) to detect and isolate male cells. The DNA profiling following FISH/LMD, however, requires a minimum of seventy-five diploid cells in the 50 µL of in-tube PCR reaction [1]. Laser capture microdissection (LCM) is an effective method for the isolation of low abundant cells from biological mixtures, such as postcoital vaginal swabs and chorionic villi of abortion material [2], [3]. The difference in size and morphology of sperm compared to vaginal epithelium makes LCM an ideal method to isolate sperm from sexual assault cases. Further, retrospective analysis of mock casework samples validated genetic profiles obtained following LCM cell isolation in many cases [4], [5], [6], [7].

Genetic profiling also requires an abundance of sample in order to generate complete allelic profiles. We have previously demonstrated the utility of on-chip low volume PCR (LV-PCR) to generate genetic profiles from very low abundant samples. In these studies, as few as three buccal cells isolated from a mixture of cells was sufficient to generate a complete genetic profile using the ABI Identifiler® kit [8]. Here we combine LCM with LV-PCR to isolate and genotype sperm cells. LCM and LV-PCR proved more sensitive that in-tube PCR, generating complete DNA profiles from as few as 15 sperm. Retrospective analysis of a DNA casework study confirmed the ability of LCM and LV-PCR to isolate and genotype spermatozoa isolation from complex mixtures.

## Results

### Sensitivity of on-chip LV-PCR

**Figure 1:**
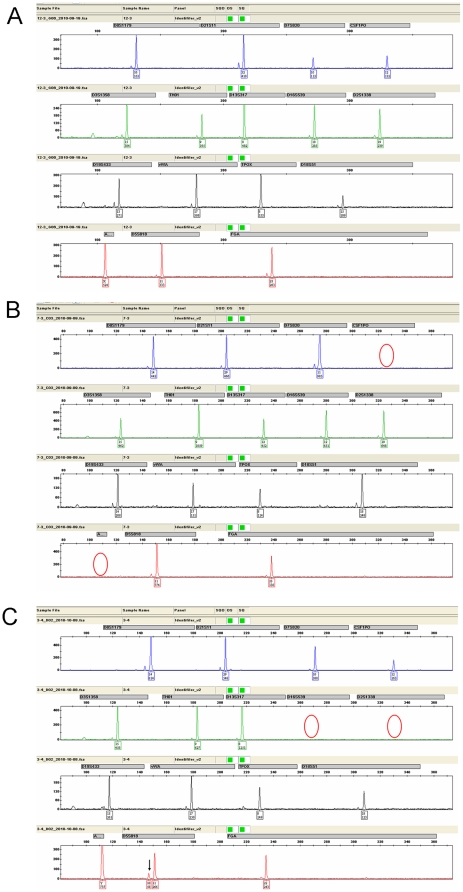
Haploid-type electropherograms derived from a single sperm. Panels A, B and C indicate electropherograms of replicate 1, 2 and 6 listed in Table 2. Red circles indicate allele dropout, arrows indicate allele drop-in. Allele call and peak height are shown under each peak.

To determine the sensitivity of on-chip LV-PCR, genetic profiling of a sperm sample was repeated multiple times using decreasing number of sperm (sperm counts analyzed ranged from thirty to one cell). Twenty replicates were performed for each group. Results of the sensitivity study are listed in Table 1. Complete profiles generated by the Identifiler® kit were obtained in each of the 20 replicates (100%) when the assay was performed on thirty sperm cells. When the number of cells analyzed was reduced to 20 or 15, the number of complete allelic profiles was reduced to 18 (90%) and 16 (80%), respectively. Allelic dropout (ADO) increased in relation to the decreased number of spermatozoa. These data identify that LV-PCR is a powerful tool for genetic analysis of sperm samples. In fact, LV-PCR performed on just a single sperm cell determined 13–16 loci of the haploid-type profile in 10 of 20 assays (50%; Table 1). Each haploid profile is listed in Table 2. Haploid-type electropherograms of replicate 1, 2 and 6 are shown in Figure 1 A, B and C.

**Table 1:** Number of genotyped loci following on-chip LV-PCR and in-tube PCR reactions in relation to the amount of spermatozoa analyzed.

Number of isolated spermatozoa	Number of genotyped loci				Total(n)	Mean loci detected ± SD
	16	13–15	9–12	0–8		
1.5 µL of on-chip LV-PCR						
30	20(100%)	0(0%)	0(0%)	0(0%)	20	16.0±0.0
20	18(90%)	2(10%)	0(0%)	0(0%)	20	15.9±0.5
15	16(80%)	4(20%)	0(0%)	0(0%)	20	15.6±1.0
10	8(40%)	9(45%)	3(15%)	0(0%)	20	14.5±1.8
5	0(0%)	7(35%)	12(60%)	1(5%)	20	11.8±1.9
1	1(5%)	9(45%)	10(50%)	0(0%)	20	12.2±2.0
10 µL of in-tube PCR						
50	7(70%)	3(30%)	0(0%)	0(0%)	10	15.3±1.2
40	4(40%)	6(60%)	0(0%)	0(0%)	10	14.6±1.3
30	0(0%)	5(50%)	4(40%)	1(10%)	10	10.9±2.8
20	0(0%)	0(0%)	2(20%)	8(80%)	10	4.9±2.8

**Table 2:** Haploid analysis of STR loci from a single sperm by LV-PCR.

Replicates	1	2	3	4	5	6	7	8	9	10	11	12	13	14	15	16	17	18	19	20	Known profile
D8S1179	10	14	10	10	14	14	10	14	14	10	14	14	10	10	14	14	10	10	—	10	10,14
D21S11	32	29	29	32	29	29	32	29	29	32	29	32	—	29	*24*,29	32	—	29	—	32	29,32
D7S820	10	11	—	—	10	10	10	—	—	—	—	—	10	—	—	—	10	10	—	—	10,11
CSF1PO	12	—	12	12	12	12	—	—	12	12	10	—	10	12	10	—	—	—	10	—	10,12
D3S1358	15	15	15	15	14	15	14	15	15	14	14	14	15	—	15	—	15	14	—	*14*,15	14,15
TH01	9	9	9	9	9	9	9	9	9	—	9	9	9	9	9	—	9	9	9	—	9,9
D13S317	8	12	8	12	8	8	12	8	—	8	12	8	12	8	8	8	12	12	—	—	8,12
D16S539	10	12	10	—	—	—	10	12	10	12	12	12	10	—	—	10	10	—	12	12	10,12
D2S1338	19	19	23	23	19	—	19	19	19	23	—	—	—	—	—	23	—	19	19	23	19,23
D19S433	13	14	14	13	14	13	—	13	14	13	13	14	13	13	13	—	13	13	14	13	13,14
vWA	17	17	17	17	17	17	17	17	17	17	17	—	—	—	17	17	-	—	—	17	17,17
TPOX	8	8	8	8	8	8	8	8	—	8	—	8	8	8	8	8	8	—	8	—	8,8
D18S51	15	18	15	18	15	18	18	15	18	15	—	15	15	15	—	—	—	—	—	15	15,18
Amelogenin	X	—	X	Y	X	Y	Y	X	X	X	X	X	Y	Y	Y	Y	Y	—	X	X	X,Y
D5S818	11	11	11	11	—	*10*,11	—	11	11	—	11	11	11	11	—	11	11	—	11	—	11,11
FGA	23	23	—	22	23	22	22	—	22	22	22	*22*,23	—	23	23	22	—	23	22	—	22,23
Allelic dropout	0	2	2	2	2	2	3	3	3	3	4	4	4	5	5	6	6	7	7	7	
Allelic drop-in						1						1			1					1	

“—” indicates allelic dropout.

Italics indicate allelic drop-in.

In contrast to LV-PCR, ‘in-tube’ PCR was unable to generate complete allelic profiles when few sperm were available for analysis. When 50 and 40 sperm cells were analyzed, only 7 and 4 complete profiles were obtained from 10 repeat assays, respectively. Thirty cells were insufficient to obtain a profile of 13–16 loci. Further, the mean loci detected by LV-PCR from just 15 sperms (15.6±1.0) was greater than the average obtained following analysis of 50 sperm by ‘in tube’ PCR (15.3±1.2).tpb

### Casework Analysis

**Figure 2:**
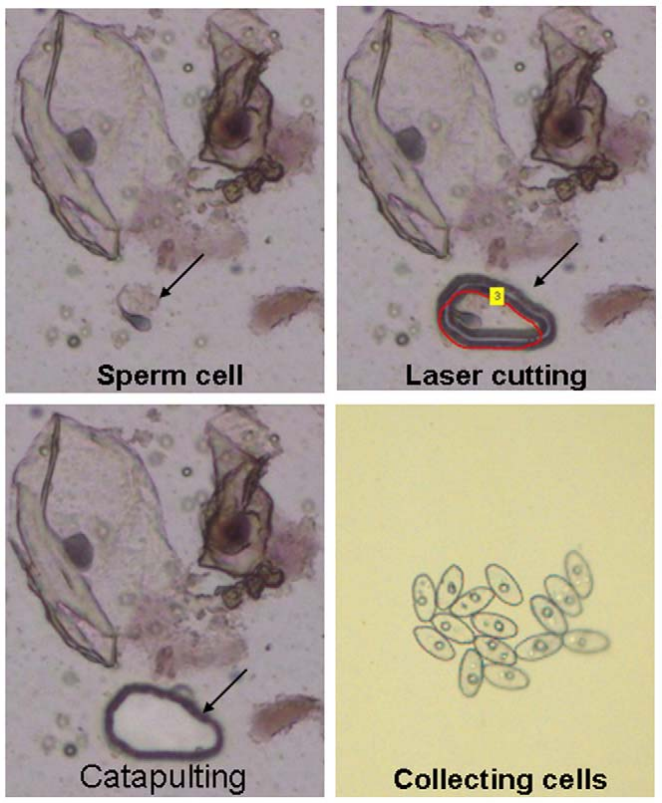
Sperm cell separation and collection by LCM. Samples were resuspended and placed on a PEN membrane slide. Sperm were identified by light microscopy (400× magnification), and removed by laser cutting. Finally, sperm cells were catapulted and collected onto a low-volume PCR slide.

Two sexual assault casework samples were obtained, in which sperm has been isolated by the epithelial lysis method and genetic profiles had been generated by standard PCR. Here, we attempted re-analysis of these samples by LCM and LV-PCR. Sperm cell separation and collection (catapulting) is shown in Figure 2. Spermatozoa are much smaller compared to vaginal epithelial cells, allowing easy identification and separation. Fifteen spermatozoa were captured and transferred onto an AmpliGrid® slide, positioned upside-down on a computer-driven manipulator platform, and analyzed by LV-PCR assay. Images acquired before and after capture clearly demonstrate the transfer process.

**Figure 3:**
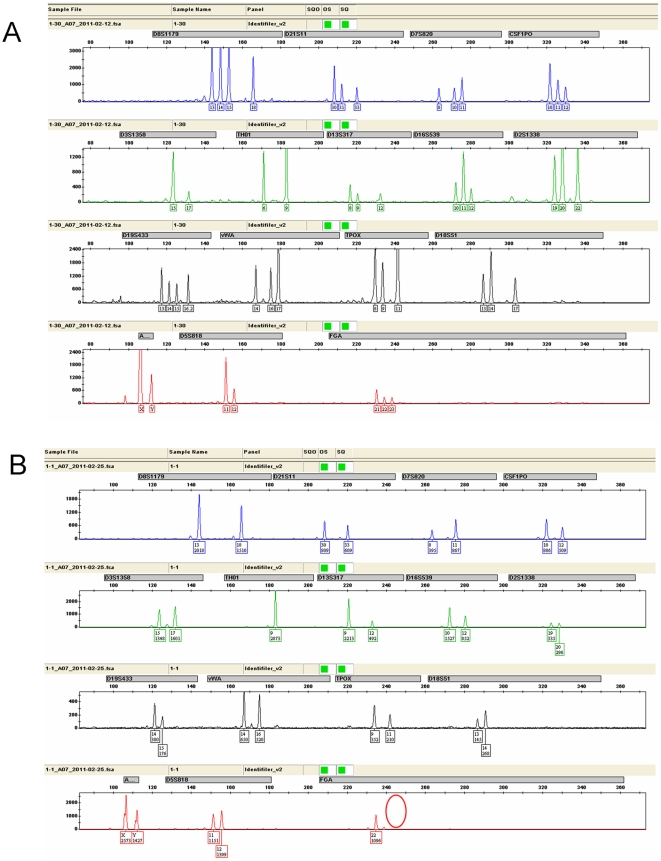
Genetic profiling of sample No. 1. (A) Preferential epithelial lysis followed by ‘in-tube’ PCR generated a mixed genetic profile from the casework sample. (B) Electropherogram profile of replicate 1 obtained by sperm isolation by LCM and profiling by LV-PCR. This method generated an individual profile with consensus to the known sample. Red circles indicate allele dropout.

Isolated sperm from sample No. 1, derived from a victim who had been sexually assaulted by a single perpetrator, was analyzed by the LV-PCR method. Whereas preferential epithelial lysis generated a mixed profile of male/female DNA (Figure 3A), LCM and LV-PCR generated a consensus single-person genotype that was in concordance with the perpetrator's profile. Detailed results are summarized in Table 3. Electropherogram profile of replicate 1 is shown in Figure 3B. Sample No. 2 was obtained from a victim of sexual assault by two male perpetrators. As observed with ‘in-tube’ PCR following preferential epithelial lysis, LCM and LV-PCR was unable to separate the two sperm samples and therefore generated a mixed profile. These data indicate that LCM and LV-PCR is efficient in sperm isolation from male/female mixtures, but current protocol does not allow for separation of two or more sperm samples from a complex mixture.

**Table 3:** Identification of a consensus single-person genotype by LCM and LV-PCR from a sexual assault casework sample.

Replicates	1	2	3	4	5	Consensus profile
D8S1179	13,18	13,18	13,18	13,18	13,18	13,18
D21S11	30,33	30,33	30,32,33	30,33	30,33	30,33
D7S820	8,11	8,11	8,8	8,11	8,11	8,11
CSF1PO	10,12	10,12	10,12	10,12	10,12	10,12
D3S1358	15,17	15,17	15,17	15,17	15,17	15,17
TH01	9,9	9,9	9,9	9,9	9,9	9,9
D13S317	9,12	9,12	9,12	12,12	9,12	9,12
D16S539	10,12	10,12	10,12	10,12	10,12	10,12
D2S1338	19,20	19,20	19,20	19,20	19,20	19,20
D19S433	14,15	14,15	14,15	14,15	14,15	14,15
vWA	14,16	14,16	14,16	14,16	14,14	14,16
TPOX	9,11	9,11	9,11	9,11	9,9	9,11
D18S51	13,14	13,14	13,14	13,14	13,13	13,14
Amelogenin	X,Y	X,Y	X,Y	X,Y	X,Y	X,Y
D5S818	11,12	11,12	11,12	11,12	11,12	11,12
FGA	22,22	22,23	22,23	22,23	22,22	22,23

## Discussion

On-chip LV-PCR using the Identifiler® kit is more sensitive than standard ‘in-tube’ PCR for generating complete gene loci profiles from sperm samples. While thirty sperm cells could not generate complete profiles by in-tube PCR, analysis of 30 cells by on-chip LV-PCR generated complete profiles in 100% of samples. Complete profiles (16 loci) were generated from as few as 15 sperm cells in 80% of samples. Further, profiling of a single sperm cell generated a minimum of 13–16 loci in 50% of samples. Data analysis of these haploid profiles indicates overlap, suggesting that full profiles may be possible under optimized conditions (Table 2). In fact, Miyazaki et al. [9] demonstrated complete haploid-type electropherograms from a single sperm following nuclear DNA amplification using improved primer extension preamplification polymerase chain reaction (I-PEP-PCR). Together, these studies support reports indicating the high sensitivity of on chip LV-PCR [8], [10], [11].

While unstained sperm can be identified by microscopy, the process is slow and laborious. Staining enhances sperm visualization and therefore increases the speed of detection and isolation. Sanders et al [5] investigation of the effect of five common histological stains on downstream PCR analysis identified that hematoxylin/eosin (H&E) outperformed the others but still resulted in lower RFU values compared with unstained specimen. A simplified hematoxylin staining method was developed here to achieve good visual identification of sperm cells with less negative effect on downstream analysis. Our results indicate that hematoxylin alone is sufficient to improve identification of sperm cells, with less impact on PCR analysis than H&E.

On-chip LV-PCR is a powerful tool for forensic DNA profiling, however the current methodology is associated with some technical drawbacks. Most prominently, allelic dropout and allelic drop-in are common. Previous reports indicate that allelic drop-in is a random occurrence, with the source of these alleles unknown [12], [13]. Our results similarly identify random allelic drop-ins in this study (Table 1 and 2). To overcome this issue, replicate analyses are performed [14]. Here, fifteen sperm cells were collected five times for each casework sample, and composite DNA profiles were generated by replicate experiments.

Additionally, LV-PCR is associated with several minor flaws. The high sensitivity of LV-PCR results in an increased risk of DNA contamination. Most contamination have been found to be a result of contamination of the laboratory setup or reagents, and as such can be avoided with careful laboratory practice [13]. To minimize the contamination risk in this study, a BSL-2 bio-safety laboratory was utilized for all cell separation and detection experiments. Further, on-chip LV-PCR does not permit replications of the same PCR product, as the entire PCR product is used for one electrophoresis run. Replicates are actually amplifications of different cell groups derived from independent reactions. Finally, gas bubbles in the reaction reagents may result in problems with the Ampligrid slide; bubbles may grow during the denaturation steps, bursting and destroying the reaction spot or merging with the adjacent spots. The problem can be minimized by the use of a pipette for small volumes (0.1–2.5 µL) and experienced manipulator.

Overall, LCM was highly effective method for the isolation of pure spermatozoa from sperm/epithelial cell mixtures in each casework sample analyzed. Compared to preferential epithelial lysis, LCM holds the clear advantage when sperm cells are in low relative abundance compared to epithelial cells. When combined with LV-PCR, LCM resulted in high sensitivity DNA profiling of sperm from a single perpetrator. Current protocols, however, cannot differentiate amongst sperm from multiple contributors. Overall, LCM and LV-PCR is a very promising method for DNA profiling of sexual assault caseworks, and may prove to be even more useful with additional technical advances.

## Materials and Methods

### Sample collection and preparation

Semen was collected for genetic analysis from a healthy volunteer. The sample was applied on tissue paper, a common carrier in casework samples, and air-dried and stored at room temperature (25°C). Vaginal swabs from two victims of sexual assault crimes were collected from routine caseworks from our laboratory. Sample No. 1 contained a sperm/epithelial cell mixture from a victim that was attacked by a single perpetrator. Sample No. 2 was obtained from a victim attacked by two perpetrators in a gang rape case. Each sample was previously processed by preferential lysis [15], and DNA was extracted by MagAttract® DNA Mini M48 Kit (Qiagen, Germany). DNA samples were amplified as described in Materials and Methods.

Tissue paper sperm specimen were cut into 0.5 cm_2_ samples and incubated in TNE buffer (10 mM Tris-HCL, pH 8.0; 10 mM NaCl; 0.1 mM EDTA) for 20 minutes at 37°C. Following centrifugation at 9000×g for 3 min and removal of the supernatant, the cell pellet was resuspended in 30 µL of TNE buffer and smeared on a polyethylene naphthalate (PEN) membrane slide (Carl Zeiss Ltd., Germany). The slide was air dried at room temperature for 5 min and stained with hematoxylin as follows: 75% ethanol for 2 min and rinsed with sterile water, hematoxylin for 1 min and rinsed with sterile water again. Following air drying at room temperature for 10 min, the slides were ready for use.

We have obtained ethics approval for our study from Ethics Committee of Institute of Forensic Science of China. Participants were recruited from staff of our institution. Written informed consent was signed by all participants involved in the study.

### Laser capture microdissection (LCM)

A PALM MicroBeam instrument (Carl Zeiss Ltd.) fitted with a 355 nm UV-laser was used for laser microdissection of spermatozoa (400×magnification). For the ‘in-tube’ PCR reactions, sperm was isolated in groups of 50, 40, 30 and 20 from the PEN slide. Cells were deposited into the caps of 0.2 mL thin-walled PCR tubes (Axygen, Union city, CA) containing 4 µL of sperm cell lysis buffer (0.1 mg/mL proteinase K (Calbiochem, Germany) containing 5 mM DTT (Merck, Germany). The samples were centrifuged at 16,000×g for 5 min to remove buffer and cells from the cap. The samples were incubated at 56°C for 40 min to lyse the cells, and boiled for 10 min to denature proteinase K. For low volume-polymerase chain reaction (LV-PCR), sperm isolated by LCM (groups of 30, 20, 15, 10, 5 or 1) were placed on an AG480F AmpliGrid® slide (Advalytix AG, Germany). Fifteen sperm cells were isolated for analysis of the casework samples. For each analysis, sperm cell lysis buffer (0.75 µL) was added to each reaction position, and then covered with 5 µL of mineral oil (Advalytix AG). Samples were incubated at 56°C for 40 min and boiled for 10 min.

### Polymerase chain reaction

Standard, ‘in-tube’ DNA Amplification was performed for 15 STR loci and Amelogenin using the AmpFlSTRs Identifiler® kit (Applied Biosystems, Foster City, CA) and a GeneAmp 9700 Thermal Cycler (Applied Biosystems). Each reaction contained 3.4 µL of sterile distilled water/DNA, 4.2 µL PCR Reaction Mix, 2.2 µL Primer Mix and 1 unit of AmpliTaq Gold DNA Polymerase (Applied Biosystems). Thermal cycling was performed as described by the manufacturer. AmpFLSTR® Control DNA 9947A (Applied Biosystems, 0.1 ng/µL) was used as positive control, and no DNA template was used as negative control.

On-chip LV-PCR was performed as described previously [8]. Positive and negative controls were performed for each AG480F AmpliGrid® slide. PCR products (total of 1.5 µL) were transferred to 10 µL of loading buffer. Five replicates of were performed for each casework sample, consisting of 15 sperm cells each. Consensus DNA profiles were generated from alleles found in triplicate among the five replicate PCR reactions [8].

### Electrophoresis

One microliter of each PCR product was denatured in 10 µL of loading buffer, composed of HI-DI™ formamide (Warrington, UK) and LIZTM-500 size standard mixture (Warrington, UK) in a proportion of 500∶1 (v/v). Electrophoresis was performed on a 3130 XL Genetic Analyzer (Applied Biosystems) using a 10 sec injection time, followed by data analysis using Genemapper ID V3.2.1 software (Applied Biosystems).
